# Improving suitability of urban canals and canalized rivers for transportation, thermal energy extraction and recreation in two European delta cities

**DOI:** 10.1007/s13280-022-01759-3

**Published:** 2022-08-24

**Authors:** E. Suzanne van der Meulen, Frans H. M. van de Ven, Pieter R. van Oel, Huub H. M. Rijnaarts, Nora B. Sutton

**Affiliations:** 1 Deltares, Postbus 85467, 3508 AL Utrecht, The Netherlands; 2 Wageningen University, Postbus 17, 6700 AA Wageningen, The Netherlands; 3 Technical University Delft, Postbus 5, 2600 AA Delft, The Netherlands; 4 Wageningen University, Droevendaalsesteeg 3, 6708 PB Wageningen, The Netherlands

**Keywords:** Bathing, Suitability Index, Thermal energy source, Urban freight transport, Urban water, Waterway

## Abstract

Canals and canalized rivers form a major part of surface water systems in European delta cities and societal ambitions to use these waters increase. This is the first assessment of how suitability of these waters can improve for three important uses: transportation, thermal energy extraction (TEE) and recreation. We assess suitability with Suitability Indices (SIs) and identify which alterations in the water system are needed to improve SI scores in Amsterdam, The Netherlands, and Ghent, Belgium. The results show spatial variability in suitability scores. Current suitability for transportation is low (SI score = 1) to excellent (SI score = 4), for TEE fair (SI score = 2) to excellent (SI score = 4), and suitability for recreation is low (SI score = 1). Suitability could improve by enlarging specific waterway dimensions, increasing discharge and clarity, and by enhancing microbiological water quality. The same methodology can be applied to optimize designs for new water bodies and for more water uses.

**Supplementary Information:**

The online version contains supplementary material available at 10.1007/s13280-022-01759-3.

## Introduction

After decades of investments in water quality, through improved wastewater treatment and stormwater management, cities are rediscovering their urban surface water as an important functional and aesthetic asset (EEA 2016b). This is also reflected by the growing attention for ‘blue space’ in urban planning and public health sciences (Dall‘O’ 2020; Georgiou et al. 2021; Smith et al. 2021; Bell et al. 2022). The urban surface water system includes many highly modified or manmade water bodies such as canals and canalized rivers. In the remainder of this article, we will use the term ‘canals’ to refer to canals and canalized rivers. While the ecological status of these waterbodies is relatively low, they provide important use functions, or ecosystem services, to citizens and businesses in European delta cities. Function-oriented design and maintenance of these water bodies will become increasingly important. Firstly, this is due to increasing use of urban surface water for human use functions, mainly driven by urban growth and regeneration, climate change, water quality improvements, and sustainability ambitions (EEA 2016b; Van der Meulen et al. 2020). Secondly, there is the ambition to provide multiple societal benefits when creating or restoring urban water courses (Wild et al. 2011; EEA 2016b).

Research in Amsterdam, The Netherlands and Toronto, Canada, has shown that demand for many use functions of urban surface water is expected to increase in the next decades (Van der Meulen et al. 2020). This increasing demand is most clear for transportation, thermal energy extraction (TEE), and different types of recreation including swimming. These trends were also reported by other authors. Janjevic and Ndiaye (2014) and Hallock and Wilson (2009) describe increasing demand for water-borne urban freight transportation, driven by road congestion and by ambitions to reduce greenhouse gas emissions from freight transport. In its New EU Urban Mobility Framework, the European Commission (2021) proposes better utilization of urban waterways for the ‘first and last mile’ in the Trans-European Transport Network (TEN-T) that is being developed until 2050. TEE from surface water is considered as one of the renewable alternatives for fossil fuel heating in Europe.^1^ The most favorable conditions for surface water based district heating are expected in urban areas close to surface water (Moller 2015). The potential of this heat source is significant: 43% of the total urban heat demand in the Netherlands can be extracted from surface water in an economically feasible way (Kruit et al. 2018) and in England, the entire heat demand of smaller urban areas along larger rivers can be obtained from the river (Department of Energy and Climate Change 2015). Increasing recreational use of urban water is illustrated by a substantial increase in the number of designated urban bathing sites in Europe in the last decade (EEA 2020). Additionally, swimming at non-designated sites is increasingly popular due to water quality improvements and higher water temperatures (EEA 2020). Swimming events in urban canals are also gaining popularity (Hintaran et al. 2018) such as the Big Jump in Ghent, Belgium,^2^ the Amsterdam City Swim^3^ and Swim-In in Leiden,^4^ The Netherlands. Several political and societal groups in Europe and the USA are advocating for more opportunities to swim in urban surface water (Ruby and Shinohara 2019; Mouchel et al. 2020).

Besides the increasing ambitions to use urban surface waters, there are ambitions to add use potential when restoring urban rivers or canals for ecological or water detention purposes. Many restoration projects in cities involve restoration of smaller urban waters (EEA 2016b). In some cases, formerly culverted or filled in waterways are recently reopened, or ‘daylighted’, such as Aarhus River in the city of Aarhus, Denmark (completed in 2015) or the historic canal Reep in Ghent (completed in 2018), Belgium. Policy documents and explorative studies indicate that more waterways will be reopened in the near future (e.g., VWW and Stad Gent 2018; MVRDV 2020). A multifunctional design that includes benefits in terms of use functions, or ecosystem services, is expected to increase financial and moral support for restoration projects (EEA 2016b) and may lead to greater sustainability (Wild et al. 2011). The European Commission considers the multiple benefits that natural water retention measures, such as open water, can provide as the primary justification for choosing green infrastructure over grey infrastructure measures (EC 2014). Among the potential co-benefits are provision of human use functions such as recreation.^5^

With increasing ambitions to use urban water for human uses, it is important to understand how to improve suitability of existing and planned water bodies for desired functions. Such insight can be used to improve design and maintenance of these waters. To date, there have been no studies described in scientific literature that clarify how suitability of urban canals can be improved for transportation, TEE and recreation. Therefore, the aim of this study is to identify which characteristics of urban canals in European delta cities should be adapted to improve suitability of the water bodies for these three use functions. To reach this aim, we first assess the suitability of urban canals for transportation, TEE and recreation in two delta cities by applying Suitability Indices (SIs) for these uses (Van der Meulen et al. 2022). Next, we analyze the impact of alterations in the water system on the SI score for each use function to identify the required alterations for improving suitability. We use the cities of Amsterdam in The Netherlands, and Ghent in Belgium, as study sites. The results of the analysis provide novel insight in how suitability of urban canals for the selected human uses can be improved.

## Materials and methods

### Method

To analyze how suitability of urban canals can be improved for transportation, TEE and recreation, we analyze which alterations are needed to improve the Suitability Index (SI) score for these use functions. We use existing SIs that were developed for the biophysical and socio-economic context of the Netherlands (Van der Meulen et al. 2022). SI Transport is targeted at urban freight transportation, which includes transport of goods to and withing the city. SI TEE is targeted at heat extraction during warm summer months, seasonal storage and use of the thermal energy in winter (See Supplementary Information S1 for a detailed explanation of the heat extraction process). SI Recreation is targeted at swimming by adults. A SI consists of parameters that describe water bodies’ characteristics that determine suitability for a single use function. The parameters are each rated by a sub-index score and sub-index scores are integrated into the SI score. Parameters include chemical, microbiological, and physical characteristics of surface waters (Table 1). Suitability is rated on a five-class scale: unsuitable (SI score = 0), low (SI score = 1), fair (SI score = 2), good (SI score = 3) or excellent (SI score = 4). Water is unsuitable if use of the function is physically impossible. See Supplementary Information (Appendix S1) and Van der Meulen et al. (2022) for more details about the SIs, including target values for the sub-scores.

**Table 1:** Characteristics of the Suitability Indices. The SI score is based on the integration of sub-scores for parameters that significantly influence the suitability for a specific use function. Target values for the sub-scores and the preconditions are given in Supplementary Information (Appendix S1)

SI characteristics	Suitability Index (SI)		
	SI Transport	SI Thermal energy extraction (TEE)	SI Recreation
Suitability based on:	Maximum possible ship size^a^ for urban freight transport	Heat extraction capacity related to typical heat demand per house (40 GJ year^−1^)	Health risk for adult swimmer
SI score classes			
4 Excellent	Large Rhine vessel	≥ 1000 houses	No or very low risk
3 Good	Barge	10–1000 houses	Low risk
2 Fair	Specialized urban vessel	10–100 houses	Moderate risk
1 Low	Smaller than urban vessel	< 10 houses	High risk
0 Unsuitable	Precondition not met	Precondition not met	Precondition not met
Parameters (correlation with sub-score):	Depth (+)	Width (+)	Depth at shore (−)
	Width (+)	Discharge (+)	*E. coli* (−)
	Air draft (+)	Water temperature (+)	Cyanobacteria (−) pH (optimum at 7–8) Clarity (+)
Precondition	Minimum depth in fairway	Minimum depth	Maximum depth
Integration method:	Minimum operator^b^	Geometric mean^c^	Minimum operator^b^

+ : higher values for the parameter lead to higher sub-score

− : lower values for the parameter lead to higher sub-score

^a^See Supplementary Information (Appendix S1) for ship dimensions

^b^*SI* = $Min\left({S}_{i=1}^{n}\right)$, where *S*_*i*_ is the sub-index score of the *i*th parameter

^c^$SI={\left(\prod_{i=1}^{n}Si\right)}^{1/n}$ where S_i_ is the sub-index score of the *i*th parameter

Our analysis consists of a simplified optimization process in four steps to identify which alterations of the SI parameters are needed to increase the SI score by at least one class. First, the SIs are applied to determine current suitability of urban canals in our study areas for transportation, TEE and recreation. Secondly, we apply a local search algorithm to explore and evaluate the impact of hypothetically higher sub-scores on the SI score for each use function. The result shows which alterations in sub-scores lead to an increase of the SI score by (at least) one class. For this first analysis, we raise sub-scores by one class. For most parameters, this is expected to be more realistic to achieve in practice than larger sub-score improvements. 4 is the maximum for sub-scores and overall SI scores (Table 1). This procedure is repeated for all parameters of the SI. The procedure is also repeated for all possible combinations of two or more parameters. Finally, we indicate the order of magnitude of the required changes in the parameter values to reach a higher sub-score. To this end, we compare the average values of the parameters with the required values that lead to a higher sub-score. The calculation of the sub-scores and overall SI scores is done with ArcGIS and MS Excel software. Sub-scores are calculated by Boolean expressions that compare the parameter values to the scoring criteria. Overall SI scores are calculated by applying the integration method that is defined per SI (see Table 1).

### Study areas

The cities of Amsterdam and Ghent are selected as study sites because they provide a comparable topographic, biophysical and socio-economic context, and an extensive network of canals and canalized rivers. Conducting the analysis in two cities, instead of one, will broaden the validity of this study. It is important that the study areas have a comparable climate, socio-economic context and geography because the SIs are context specific with respect to the parameters that are reviewed and their scoring criteria. Amsterdam and Ghent are low lying delta cities in North-western Europe with a moderate sea climate (Table 2). The two cities have highly managed surface water systems dominated by canals and canalized rivers. This provides ample opportunities to assess suitability of different types of canals and canalized rivers. Both cities face population growth and densification (Table 2). Together with drivers like water quality improvements and climate change, this increases ambitions to use the surface water for uses like transportation, recreation or TEE (VWW and Stad Gent 2018; Van der Meulen et al. 2020).

**Table 2:** Characteristics of the study areas. *T*_max_ maximum temperature, *prec.* yearly precipitation (Beck et al. 2018; Copernicus Land Monitoring Service, s.a.; EEA 2021a; Federale Overheidsdienst Binnenlandse Zaken 2018; Gemeente Amsterdam 2018a, b; KMI, s.a.; Metropoolregio Amsterdam et al. 2018; Stad Gent, s.a.)

Characteristics	Amsterdam (The Netherlands)	Ghent (Belgium)
Geography^a^	Low land delta 25–35% water + adjacent lakes and waterways	Low land delta 2.1% water
Surface water	Rivers, canals, shallow and deep lakes, ponds	Rivers, canals, deep lake, ponds
Climate	Moderate sea climate T max. 5.8 °C (Jan) to 22.1 °C (Aug) Prec. 897 mm	Moderate sea climate T max. 6.7 °C (Jan) to 23.4 °C (Jul-Aug) Prec. 876 mm
Population density	3850 per km^2^ (total surface area) 5178 per km^2^ (land area)	1643 per km^2^ (total surface area)
Population 2018	854 047	259 570
Projected population growth	+ 18% by 2040	+ 3% by 2030

^a^Percentages refer to the part of the surface area that is covered by water

The increasing ambitions for water-borne transportation are mainly targeted at local freight transportation, such as building materials or waste; ambitions to increase water-borne public transport are limited (VWW and Stad Gent Gent 2018; Van der Meulen et al. 2020; VRT 2021). Water-related recreation includes, amongst others, swimming, canoeing, fishing and boating. In both cities, water-related recreation is important as a social, cultural and economic factor for both inhabitants and visitors. Demand for swimming in urban canals is increasing, even though they are not designated as bathing waters (VWW and Stad Gent, 2018, PZC 2020; Van der Meulen et al. 2020). Demand for TEE is especially increasing in Amsterdam, where the water is used for both heating and cooling (Van der Meulen et al. 2020).

**Fig. 1:**
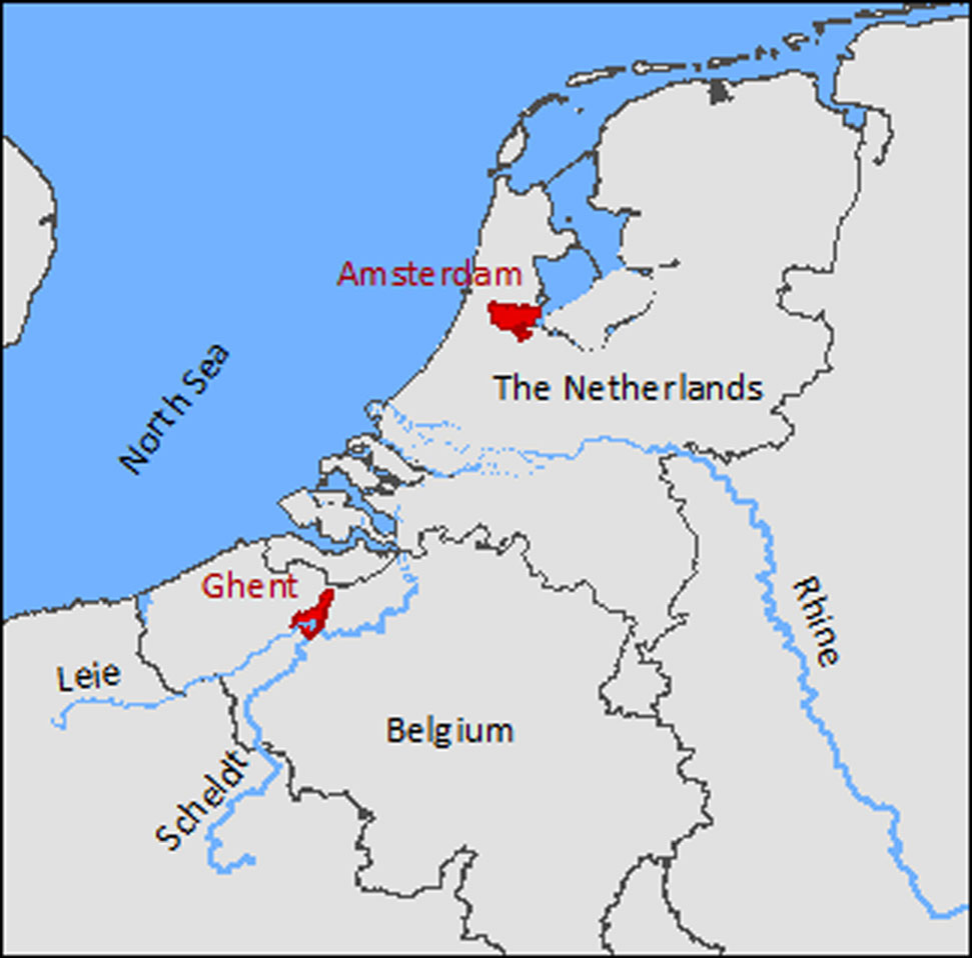
Location of Amsterdam and Ghent in the Rhine- and Scheldt deltas

**Fig. 2:**
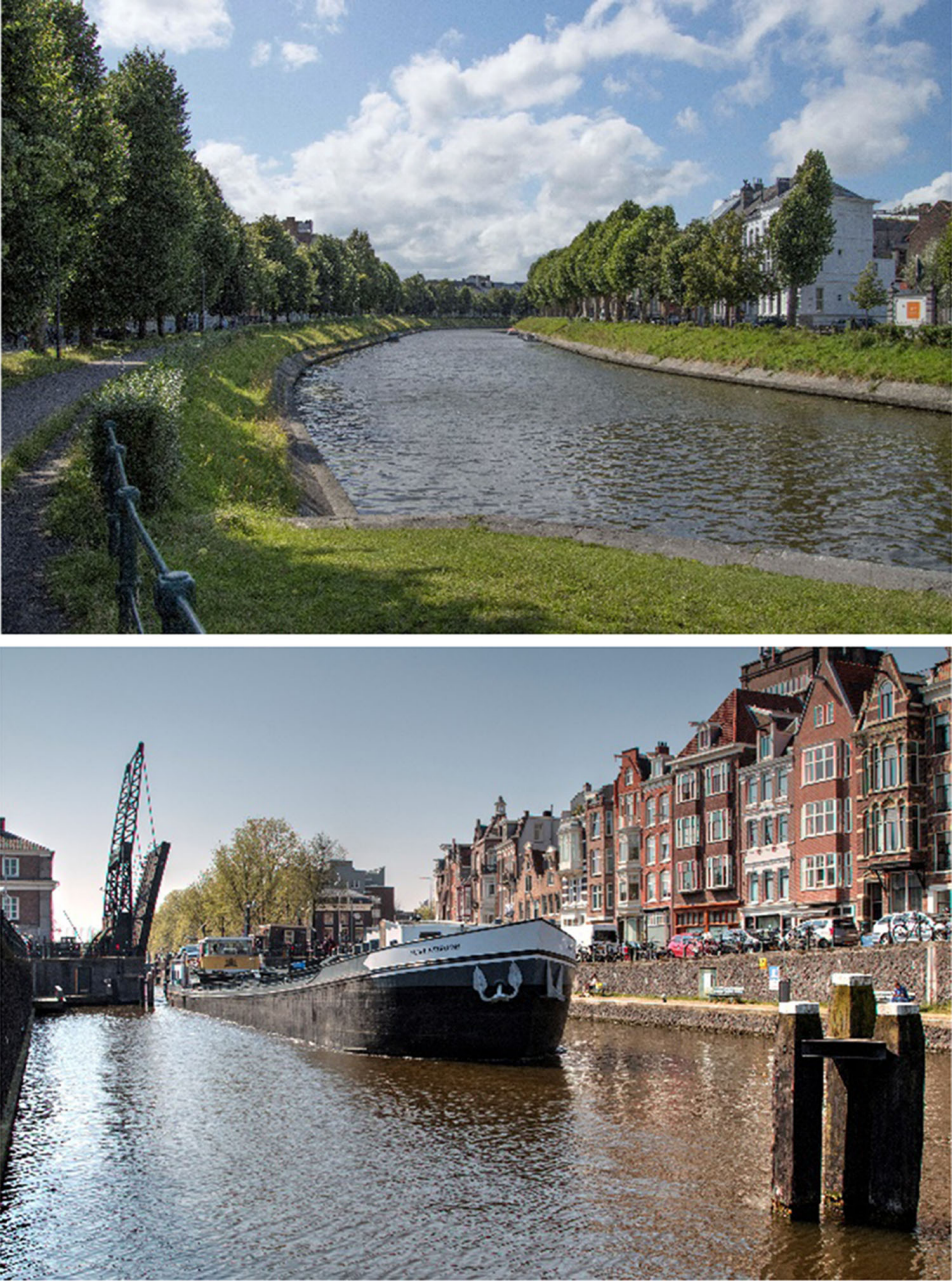
Most canals and canalized rivers have vertical quay walls (bottom, Schippergracht in Amsterdam), some have sloping banks with hard bank protection (top, Coupure in Ghent). Pictures by Marc Brink

Amsterdam is located in the Rhine-delta (Fig. 1). Canals and rivers, such as Amstel River, transport water to the city. A large canal discharges into the North Sea, 25 km west of the city; this canal is also the main artery of the sea harbor. A fine network of smaller canals, with the highest network density in the historic city center, covers the entire city. Ghent is located in the Scheldt-delta (Fig. 1). Surface water enters and leaves the city through canals and the rivers Leie and Scheldt that confluence in the city center. Other major waterways are the ring canal around the city and a large canal that leads from the harbor in the north of the city to the Scheldt estuary, approximately 30 km north of the city center. Several smaller and larger canals connect the main waterways. Most canals and canalized rivers in both cities have vertical quay walls and some have a sloping bank with paved or unpaved slope and hard bank protection (Fig. 2). Water levels in both cities are managed at a fixed level in almost all parts of the water system included in this research (VWW and Stad Gent 2018; unpublished water levels map from AGV).

Respectively 99.9% and 96.6% of waste water is collected by the sewer system in Amsterdam and Ghent (Waternet 2016; VMM 2022), more than the average of 90% in Europe (EEA 2021b). In 75% of Amsterdam, wastewater and storm water are collected separately (Waternet 2016). In these separated systems, storm water is discharged directly into surface water and wastewater is transported to wastewater treatment plants (WWTPs). Area’s developed before 1923, in the city center, have a combined sewer system that transports all water to WWTPs (Waternet 2016). In Ghent, most areas have a combined wastewater and storm water sewer system (Sumaqua 2021). In both cities, combined sewer overflows occur during heavy rainfall.

Chemical and ecological water quality of the Amsterdam canals and river system is “bad” to “moderate” according to the European Water Framework Directive water quality classification (AGV 2018; Ministerie van Infrastructuur en Milieu 2018). While fecal pollution and eutrophication are locally declining, chemical pollution remains persistent (AGV 2015). In Ghent, the physico-chemical status is “bad” to “insufficient”, mainly due to eutrophication, and the ecological status is “bad” to “moderate” (Bekkensecretariaat Bekken van de Gentse Kanalen 2016).

### Data for Suitability Index analysis

Data are obtained from Dutch and Belgium water management authorities. Most data for Amsterdam are provided by the regional water authority Amstel, Gooi en Vecht (AGV) and the Municipality of Amsterdam. Most data for Ghent are provided by, or obtained from, the municipality Stad Gent, Vlaamse Waterweg (VWW), and Vlaamse Milieu Maatschappij (VMM).

The surface water system is divided into Hydro Units (HUs) by local authorities. HUs represent sections in a waterway with unique properties. If data are available for larger spatial units than the original HUs, they are merged into new HUs. In Amsterdam, length of the HUs varies between approximately 50 m and 300 m; in Ghent, length varies between approximately 100 m and 3 km. The analyses are performed for HUs with sufficient data. SI scores for transportation are calculated for respectively 319 and 41 HUs in Amsterdam and Ghent, and for TEE in 296 (Amsterdam) and 42 (Ghent) HUs. Due to the known high spatial variability of some water quality parameters, SI scores for recreation are calculated for point locations (12 locations in Amsterdam and 7 in Ghent). The available datasets in Amsterdam lack information for some of the largest national waterways that cross the city and the adjacent sea harbor. In Ghent, we also exclude the sea harbor outside the primary city area to support comparison with Amsterdam and to focus on urban waters.

For temporally variable parameters, we use available data from a recent 4-year period. We use the (interpolated) 5th or 95th percentile of the values, in order of increasing value, at a location to ensure that the suitability score represents the minimum suitability that is valid in 95% of the time. The analyses were first performed for Amsterdam with a dataset covering the period 2016–2019. To broaden the validity of this study, Ghent was later added as second study area. The available dataset covered 2018–2021, which is partly overlapping with the period covered by the Amsterdam dataset. In Amsterdam, most water quality parameters are measured at least monthly, and we include locations with at least three years of data. In Ghent, such frequent monitoring is only available for three locations. Therefore, we also include locations with at least 3 days of water quality data. For discharge in Amsterdam, we use the 25th percentile because the 5th percentile is near zero in many locations due to flow direction variation. Because of the seasonal character of the activities, only data for the summer months April to September are used for SI Recreation, and data from the three warmest months are used for SI TEE.

Available datasets lack information for particular parameters. Cyanobacteria samples are only taken and analyzed in case of a suspicion of cyanobacteria problems, e.g., visible algae blooms. For locations in Amsterdam where cyanobacteria data are lacking, water managers from AGV confirm that cyanobacterial blooms are absent or very rare. We therefore assume that cyanobacteria are not present. In Ghent, regular observation of potentially high concentrations only takes place at the recreational canal Watersportbaan. As the cyanobacteria registration contains no notifications for this water body, we assume that cyanobacteria are not present. For all other locations we assign a sub-score of 1 because the online register and local experts indicate that cyanobacterial blooms occur in many parts of the water system, so potential high concentrations cannot be ruled out. For clarity, the Ghent dataset includes less than three days of data outside three intensively monitored locations. However, at all locations the sub-score for this parameter is 1 based on available data. Because of the limited spatial and temporal variability of this parameter, we assume that the sub-score for clarity is always 1 in Ghent. Discharge data are not available for HUs in our analysis in Ghent. Therefore, we made indicative calculations for SI TEE with four scenarios for discharge. To retrieve depth information specifically for 1 m from the shore and at the deepest point in the waterways’ profile, additional field measurements were performed by means of a lead line in Amsterdam and Ghent. See Supplementary Information (Appendix S2) for more details on data and the complete dataset that was used to calculate SI scores.

## Results and discussion

### Suitability and required interventions to improve the SI scores

#### Transport

**Fig. 3:**
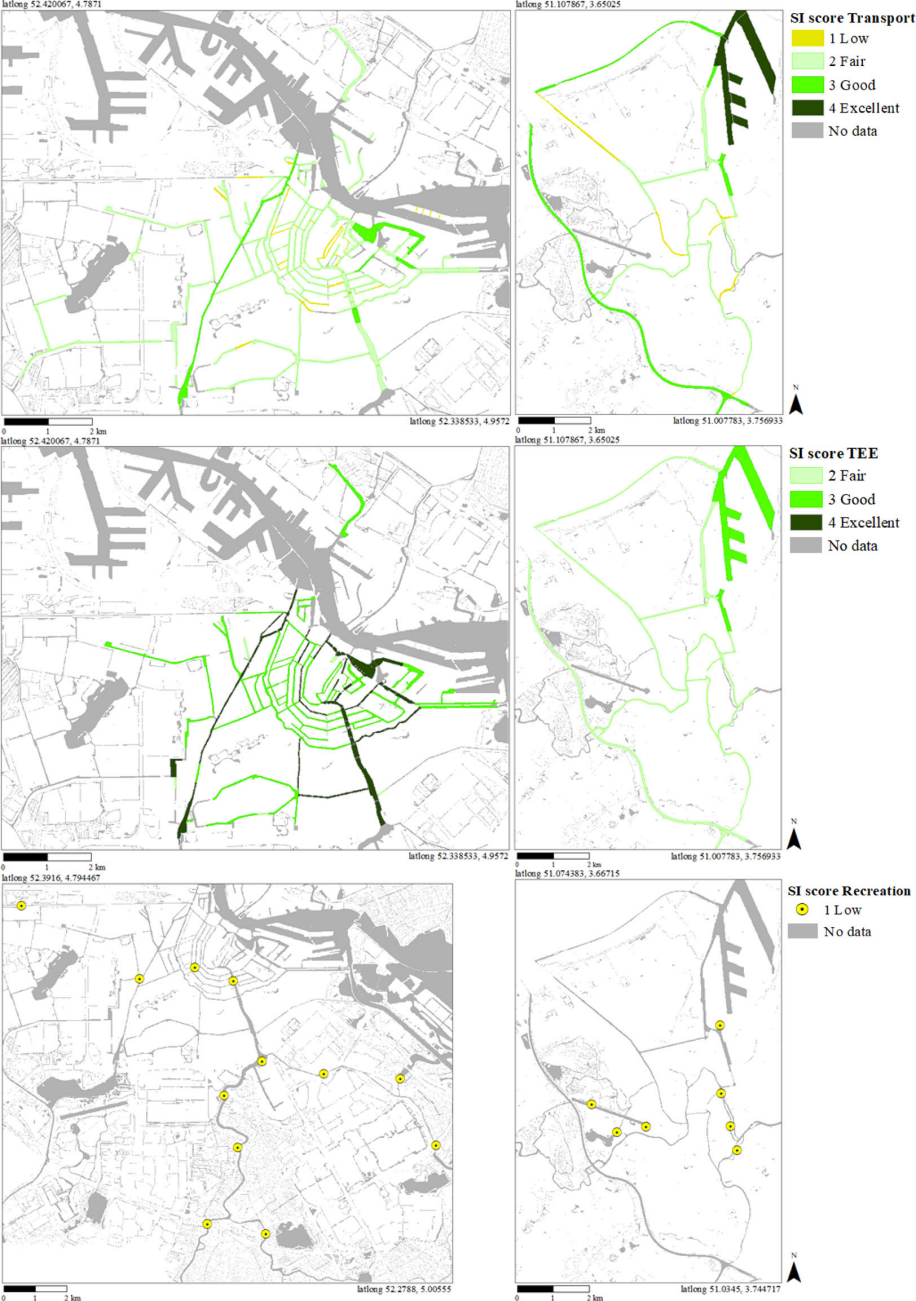
SI scores for Transport, TEE and Recreation in Amsterdam (left) and Ghent (right). SI TEE scores for Ghent are hypothetical scores based on the assumption that discharge sub-scores are 1 everywhere. Maps for Amsterdam were previously published, with minor alterations, in Supplementary Information in Van der Meulen et al. (2022)

In Amsterdam, suitability for transportation is fair in most waterways (Fig. 3). Suitability is low (SI score = 1), fair (SI score = 2) or good (SI score = 3) in respectively 17% (55 HUs), 76% (244 HUs), and 6% (20 HUs) of the HUs. Most HUs with low suitability represent short sections within waterways with fair suitability in the city center. In Ghent, suitability for transportation of most waterways within the city center is low (SI score = 1) or fair (SI score = 2), like in Amsterdam (Fig. 3). Outside the center, suitability ranges from low (SI score = 1) to excellent (SI score = 4). The ring canal around the city and a few sections in the southern part of the harbor have a good suitability (SI score = 3). Canals with excellent suitability (SI score = 4) are all located in the sea harbor in the north.

**Fig. 4:**
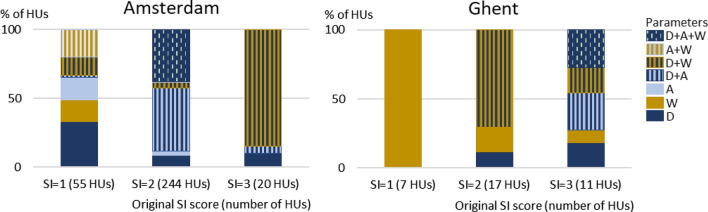
The percentage of HUs where improving the original SI score for Transport by one class requires increasing the sub-score for depth (*D*), width (*W*) and/or air draft (*A*) by one class. (Combinations of) parameters are not shown if raising their sub-score is not required or not sufficient to increase the SI score

For all HUs with a SI score lower than 4, the impact of raising sub-scores by one class for depth, width and air draft is assessed. In Amsterdam, improving suitability for transportation usually requires higher sub-scores for multiple parameters (Fig. 4). HUs with low suitability (SI score = 1) are an exception to this. In 65% of these HUs, the SI score can be raised by improving just one parameter; elsewhere, a combination of increased depth, width and/or air draft is required. On average, the required increase is 0.3 m for depth, 1.9 m for width and/or 0.5 m for air draft in HUs where these parameters are limiting the SI score to 1. To increase suitability from fair (SI score = 2) to good (SI score = 3), both depth and air draft need to be increased in most cases (Fig. 4). In many HUs (38%), mostly in the inner city (Supplementary Information, Figure S1), width needs to be enlarged as well. On average, the required increase is 1.0 m for depth, 5.7 m for width and/or 1.7 m for air draft in HUs where these parameters are limiting the SI score to 2. For HUs with good suitability (SI score = 3), a combination of depth and width is most frequently (in 85% of the HUs) required to improve suitability further (Fig. 4). On average, the required increase is 2.6 m for depth and 14.8 m for width in HUs where these parameters are limiting the SI score to 3.

In contrast to Amsterdam, air draft is not limiting suitability in HUs with low or fair suitability in Ghent (Fig. 4). Some bridges are lower than 4 m, the lower boundary for air draft sub-score 3, but these are movable bridges. To raise suitability from low (SI score = 1) to fair (SI score = 2) always requires increased width, mostly at locks (Fig. 4). On average, the required increase is 1.6 m in HUs where width is limiting the SI score to 1. Improving suitability from fair to good (SI score = 3) requires increased depth and width; in a few cases improving just one of these is sufficient (Fig. 4). On average, the required increase is 1.0 m for depth and 5.9 m for width. In some HUs, the SI score will increase if width is enlarged at specific spots, usually engineering structures like bridges or locks; in other cases, the entire profile of the waterway should be enlarged. To improve suitability from good (SI score = 3) to excellent (SI score = 4) in the ring canal requires larger depth, in the southwestern section in combination with air draft and in the northern section in combination with width at bridges and locks and sometimes also air draft (Fig. 4). On average, the required increase is 2.4 m for depth, 11.3 m for width and 3.4 for air draft in HUs where these parameters are limiting the SI score to 3.

The results show that suitability for transportation can improve by increasing depth, width, and/or air draft, with large variations within and between the study areas. Larger water depth may be achieved by dredging or by removing or adjusting under water structures. Water level management can be considered in transects where water level is controlled by locks or sluices. However, apart from short sections with low suitability in Amsterdam, the required increase of depth is quite large (> 1 m) which may not be feasible in practice. Especially in Amsterdam, increasing water levels through water level management is not an option as suitability will only improve in many HUs by increasing both water depth and air draft. Raising the water level to increase water depth results in a decrease in air draft. To increase air draft, an alternative to raising or removing fixed bridges is to replace them by movable bridges. The benefits of movable bridges are illustrated by the fact that in Ghent, air draft is less frequently limiting the suitability score for transportation compared to Amsterdam. When assigning a suitability sub-score for air draft, movable bridges are treated the same as open water. However, depending on the operation practice their presence may lead to waiting times and thus longer travel time. Travel time is an important performance indicator for logistic operations (Van Duin et al. 2017) and therefore this aspect should be taken into account when a detailed assessment for investment decisions is made. The requirement to increase width sometimes involves widening of the channel, sometimes engineering structures like locks and bridge piers are limiting suitability. Widening of the channel (on average 2–15 m) may be challenging since space in an urban setting is limited, but it may be feasible at the expense of other uses of space like parking lots or sidewalks. Alterations to quay walls and engineering structures like bridges or locks may also be limited by technical constraints.

#### Thermal energy extraction

In Amsterdam, suitability for TEE is mostly good (Fig. 3). Suitability is fair (SI score = 2), good (SI score = 3) or excellent (SI score = 4) in respectively 2% (*n* = 7), 73% (*n* = 216) and 27% (*n* = 73) of the HUs. Suitability in Ghent is uncertain due to a lack of discharge data. Calculations with different hypothetical discharge sub-scores show that, like in Amsterdam, suitability for TEE is expected to be at least fair (SI score = 2). If we assume the worst-case scenario in which the sub-score for discharge is 1 everywhere, suitability for TEE is fair (SI score = 2) in most canals in and around the city center (Fig. 3); suitability is good (SI score = 3) in the largest canals in the south part of the sea harbor area.

**Fig. 5:**
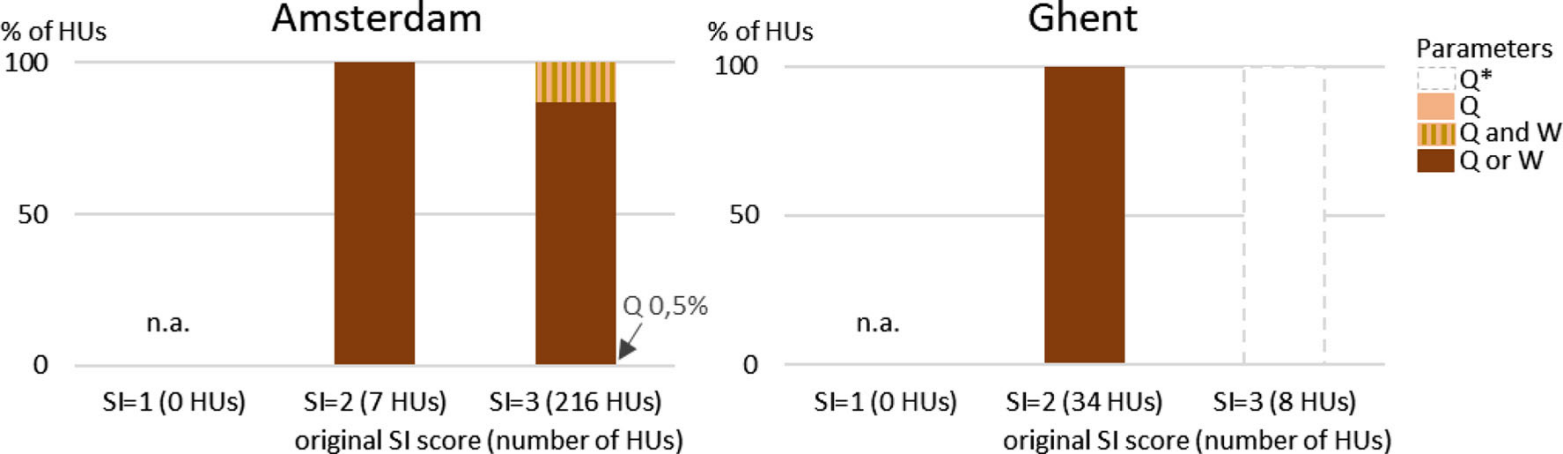
The percentage of HUs where improving the original SI score for TEE by one class requires increasing the sub-score for discharge (*Q*) and/or width (*W*) by one class. In Ghent HUs with originally SI score = 3, increasing the SI score requires increasing the sub-score for discharge by 2 classes. (Combinations of) parameters are not shown if raising their sub-score is not required or not sufficient to increase the SI score. *n.a.* not applicable

For HUs with an original SI score lower than 4, the impact of raising sub-scores by one class for width and/or discharge is assessed. The sub-score for temperature is already 4, the maximum sub-score, in all HUs in both cities and therefore does not limit suitability. In most HUs in Amsterdam, improving suitability for TEE requires increasing either width or discharge, it does not matter which one of the two. For HUs with fair suitability (SI score = 2), improving one of these parameters always results in a higher SI score (Fig. 5). In 13% of the HUs with good suitability (SI score = 3), the SI will only increase if both width and discharge are improved (Fig. 5). Required changes for width and discharge are substantial. E.g., current width and discharge values in Amsterdam HUs with good suitability are on average 27 m (average sub-score width = 3) and 0.14 m^3^/s^−1^ (average sub-score discharge = 3). To raise the sub-scores for these parameters to 4, values need to increase to ≥ 100 m or ≥ 0.3 m^3^/s^−1^ (see also Supplementary Information, Appendix S1).

In Ghent, we use different scenarios for discharge sub-scores to deal with the lack of discharge data. If the discharge sub-score is assumed to be 1, improving suitability from fair (SI score = 2) to good (SI score = 3) requires higher discharge or width, like in Amsterdam (Fig. 5). Current width in HUs with fair suitability is on average 40 m (average sub-score width = 3), assumed discharge is < 0.003 m^3^/s^−1^ (sub-score discharge = 1); to raise the sub-scores by one class requires width to become ≥ 100 m or discharge > 0.003 m^3^/s^−1^. To raise suitability from good (SI score = 3) to excellent (SI score = 4), discharge should be raised at least two classes, which requires a value of > 0.03 m^3^/s^−1^ (Supplementary Information, Appendix S1). In other discharge scenarios, the required alterations in the water system vary between higher discharge, discharge *and* width, and either discharge *or* width.

These results show that in most waterways, increasing width or discharge is required to improve suitability for TEE. Since width needs (on average) a factor 3 increase to achieve a higher SI score and space in limited in a city, increasing discharge may be a more feasible option. This may be implemented in specific waterways where demand for thermal energy is higher than the potential supply. This measure was also suggested in a case study by Van der Brugge et al. (2022). Since European delta cities like Amsterdam and Ghent have highly managed water systems, it may be feasible to direct more water to specific waterways to increase flow. However, this opportunity may be limited during dry periods, as illustrated by reported temporal water shortages during summer in one of the canals that feeds the surface water system of Ghent (Sumaqua 2021). Hydrological droughts are projected to become more frequent and severe in many parts of Europe including, but less strongly, in the temperate North-western Atlantic region (EEA 2017). Alternatively, it might be sufficient to install a surface water circulation system rather than supply water from an external source to regenerate the temperature of the surface water after heat extraction. Local hydrological assessments can give more insight into the opportunities and constraints for managing discharge, which in a highly managed water system is strongly impacted by policy choices and prioritization of water use (Sumaqua 2021).

#### Recreation

Suitability for recreation is low (SI score = 1) at all assessed locations in Amsterdam and Ghent (Fig. 3). The impact of raising sub-scores by one class for *Escherichia coli* (*E. coli*), cyanobacteria, pH, clarity, and depth is assessed.

**Fig. 6:**
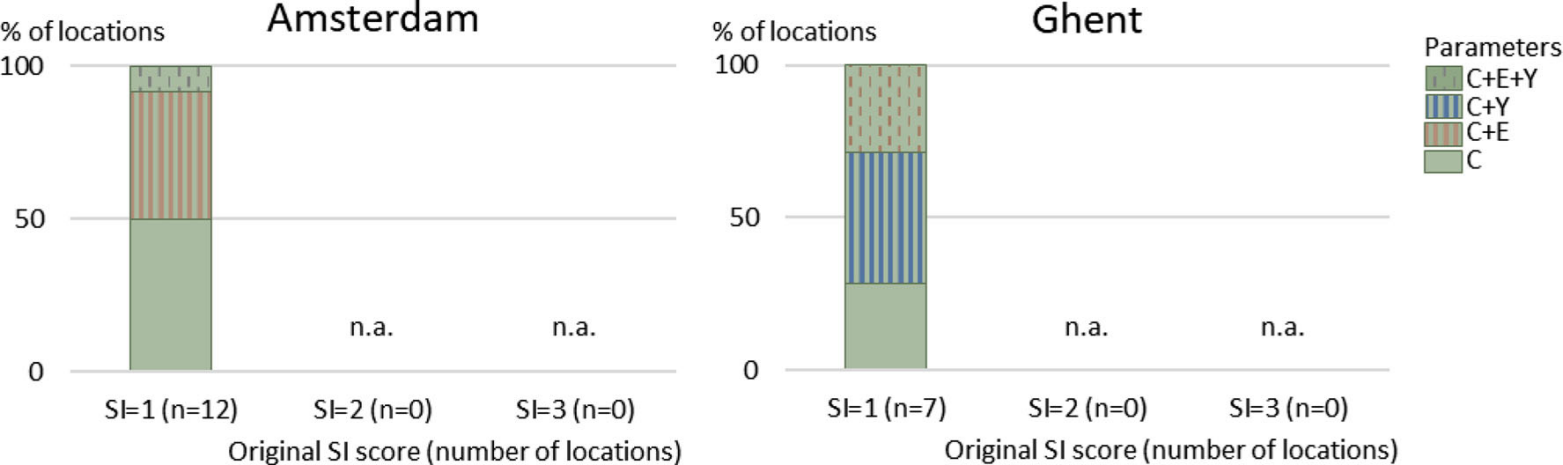
The percentage of HUs where improving the original SI score for Recreation by one class requires increasing the sub-score for clarity (C), *E. coli* bacteria (E) and/or Cyanobacteria (Y) by one class. Parameters, or combinations of parameters, are not shown if raising their sub-score is not required or not sufficient to increase the SI score. *n.a.* not applicable

In both cities, the SI score can only be raised from low (SI score = 1) to fair (SI score = 2) by improving the sub-score for clarity alone or in combination with other parameters (Fig. 6). In Amsterdam, improving clarity only is sufficient in 50% of the locations; these locations are all situated outside the city center (Supplementary Material, Figure S2). At five of the 12 locations (42%), the SI score will only improve by increasing sub-scores for clarity and *E. coli* (Fig. 6). At one of these locations, the sub-score for cyanobacteria also needs to be improved to raise the SI. In Ghent, raising the sub-score for clarity alone leads to higher SI scores at two of the seven locations. These are located in a canal that is designated for recreation but not designated as bathing water (Supplementary Information, Figure S3). Elsewhere, improving suitability also requires better sub-scores for cyanobacteria, and in two cases also for *E. coli*. If cyanobacteria are monitored at all locations, some may receive a higher sub-score for this parameter.

On average, the required increase in clarity is > 0.70 m in Amsterdam and > 0.75 m in Ghent to reach at least 1.2 m clarity (measured as secchi disk transparency), which is required for sub-score 2. *E. coli* concentrations need to be reduced by approximately a factor of 2.9 (Amsterdam) and 1.6 (Ghent), from on average 3020 cfu 100 ml^−1^ and 1190 cfu 100 ml^−1^ respectively. At one location in Amsterdam, cyanobacteria need to be reduced by approximately 11%.

Additionally, we also assessed which changes in sub-scores are needed to further increase the SI score to 3 (good) because improving sub-scores more than one class may be feasible for some water quality parameters. In Amsterdam, this requires larger improvements for clarity, *E. coli* and cyanobacteria and additionally, at some locations depth should be reduced. The same holds for Ghent. While in Amsterdam the sub-score for pH is never lower than 3, in Gent pH should be improved at one location to achieve a SI score of 3. There is a clear difference between the recreation canal Watersportbaan and the other waterways. At Watersportbaan, achieving good suitability (SI score = 3) only requires raising sub-scores for clarity to 3.

The fact that clarity is limiting suitability for recreation at all locations may indicate that improving this parameter will be difficult. However, due to Covid restrictions that resulted in less boat traffic, clarity in Amsterdam canals improved from less than 1 m to more than 1.5 m at some locations in March–April 2020 (BNNVARA 2020; H_2_O 2020). The same phenomenon was observed in canals in Venice, Italy (The Guardian 2020). This indicates that boating restrictions may help to improve clarity locally. Further local assessment is needed to identify the cause(s) of limited clarity. Besides resuspension of sediment by navigation, it may be caused by inflow of turbid wastewater or storm water, phytoplankton, or aquatic humus (Davies-Colley et al. 2003). An example from Vienna, Austria, shows how restoration measures that reduced eutrophication of an urban lake resulted in higher clarity (Teubner et al. 2020). If it is not feasible to improve clarity, an alternative solution may be to assess and take away the risks that are not visible due to limited clarity such as sharp objects under water. This approach is implemented by organizers of the Big Jump swimming event in Ghent (Hannes Cosyns, personal communication, 8 juli 2021) and before the swimming season by water management organization Waternet in Amsterdam (Liesbeth Hersbach, personal communication). For reducing *E. coli* to improve its sub-score, permanent or intermittent sources of fecal pollution should be identified. Important potential sources of fecal pollution are WWTP discharge, CSOs, sewer system failures, or animals like birds (EEA 2016a). If the sources are taken away, *E. coli* sub-scores can immediately raise more than one class. The potential effectiveness of measures to reduce bacteria concentrations can be simulated with models that take into account dilution and bacterial decay (Von Sperling and Von Sperling 2013). General strategies to prevent cyanobacterial blooms include reduction of external and internal nutrient inputs, artificial mixing of stagnant water, increasing water flow to avoid stagnant water and reduce residence time, and biological control measures such as introduction of zebra mussels that filter out cyanobacteria (Huisman et al. 2018).

### Compatibility of the required improvements for transport, thermal energy extraction and recreation

The required changes in the water system to increase suitability for transport, TEE, and recreation are compatible with each other. The required changes per use function do not have an adverse effect on the SI sub-scores for the other functions. For example: improving suitability for recreation requires increased clarity. This is not a parameter that determines the SI score of TEE or transportation (see Table 1), thus changes will not affect suitability for these uses. There is one exception: if depth is enlarged to optimize for transportation, this may affect suitability for recreation. Especially when deepening close to the shore, the sub-score for depth in SI Recreation may decline. Opportunities for synergy exist as well; TEE and transport both benefit from enlarged width, although TEE needs a larger increase to achieve a higher sub-score than transport. Increasing discharge is beneficial to TEE and indirectly also to recreation as it may potentially improve water quality parameters like cyanobacteria concentration.

While the required alterations in the water system are mostly compatible between the three analyzed use functions, simultaneous use in space and time may lead to conflicts. For example, shipping may induce reduced clarity and lead to collision risk to swimmers. Some trade-offs can be managed by temporal and/or spatial delineation of conflicting uses (Day et al. 2008; Gemeente Amsterdam 2016). This strategy may also be implemented when human uses conflict with protection of ecological quality of aquatic ecosystems.

### Novel insights

This study provides new insight into the suitability of urban canals for transportation, TEE and recreation in two delta cities, and identifies opportunities to enhance the suitability. Whereas other studies have provided insights into the feasibility of different urban water transportation concepts (Maes et al. 2012; Van Duin et al. 2017), this study shows how the canals themselves can be optimized for transportation of goods to and within the city. Maes et al. (2012) stated that scaling up of water-borne urban freight transportation is hampered by the dimensions of the existing infrastructure. In this study, we specify which dimensions have to be altered to improve suitability for transportation in Amsterdam and Ghent. Maes et al. (2015) state that adjusting vessels to reduce the required water depth and air draft is a better solution than expanding water capacity for transportation because of issues related to physical limitations, costs and potential environmental impact. We expect that these issues will be less of a bottleneck when improving suitability of short sections in the canal network. Our results show that in both study areas, the entire canal and river network can become suitable for existing special urban freight vessels by improving depth, width and/or air draft in short sections with low suitability.

This study shows that smaller to larger urban canals in two delta cities have a fair to excellent suitability for TEE. Additionally, our analysis shows how suitability of these waterways for TEE may be improved by increasing width or discharge. These insights complement previous studies on surface water as a heat source that have shown the significant potential of heat extraction from inland waters at the regional or national scale, often targeting larger rivers, canals and/or lakes (e.g., Department of Energy and Climate Change 2015; Lund and Persson 2016; Kruit et al. 2018).

This is the first study that shows which characteristics of urban canals should be altered to improve suitability for primary contact recreation. To improve the low suitability in Amsterdam and Ghent to a fair level, clarity should be increased, often in combination with a reduction of fecal pollution (for which *E. coli* bacteria are used as indicator) and/or cyanobacteria. These findings are in line with a study in a tropical urban lake (Azevedo Lopes et al. 2020) that showed that suitability for contact recreation was most limited by low visual water clarity, fecal contamination and cyanobacterial blooms. The role of fecal pollution also has been reported in analyses of gastrointestinal illness outbreaks under urban canal or river swimmers in European cities (Hall et al. 2017; Joosten et al. 2017). Studies in designated bathing water typically focus on water quality parameters only (e.g., Nagels et al. 2001; Azevedo Lopes et al. 2020). Our study in urban waterways, that are not designated bathing waters, indicates that for further improvement of the suitability for primary contact recreation to a good or excellent level, more parameters need to be considered, especially depth close to the shore.

### Limitations and suggestions for future research

We analyzed the impact of increasing sub-scores lower than 4, the maximum score. In practice, enhancing parameters that are already classified as excellent (sub-score = 4) may improve suitability for a use function. For example, SI TEE will not improve if water temperature increases since the sub-score for this parameter is already 4 in all locations. However, expected increase of water temperature of inland waters in Europe due to climate change (EEA 2017) may extend the heat extraction season and hence enforce the opportunities for TEE.

Quality of the results is influenced by the reliability of the available data to calculate the current SI scores. Subsequently, this may influence which parameters are identified as limiting the suitability. First, SI parameter values in the available datasets may differ from the actual situation in the field. For example, the dataset in Amsterdam describes the smallest width of the canal itself, not taking into account smaller sections at engineering structures like bridge piers and locks. Especially in larger waterways, sub-scores for width could be lower if detailed bathymetric data could be used. Secondly, the available data in Amsterdam and Ghent are not entirely comparable for some parameters. This applies mainly to the data on waterway dimensions since data are collected by different methods and the varying lengths of the HUs. Thirdly, most uncertainty is related to the results for TEE and Recreation in Ghent due to the lack of discharge and cyanobacteria data. Sub-scores for cyanobacteria in Amsterdam are partly based on expert judgement which also reduces reliability.

As a consequence of the abovementioned issues, parameters may be wrongfully identified as the limiting factor for suitability of a use function. However, the average required change in parameter values to reach a higher sub-score are substantial (see “Transport”, “Thermal energy extraction” and “Recreation” sections). This indicates that the general insights from this study are valid.

The results from the two case studies indicate that enhancing suitability for transportation requires locally specific alterations in the canal system. For TEE and recreation, the parameters requiring improvements are more comparable between the two cities. This may indicate that the results are also relevant to other European delta cities. The insights from this study provide a solid foundation for more detailed local assessments to support management and planning decisions, and for future research in other cities and for more use functions. To improve knowledge on the functional quality of urban canals and how suitability can be enhanced, we advise to start targeted monitoring and/or modeling campaigns in multiple cities.

To support planning of urban water use, trade-offs between human uses, and between human uses and ecological conditions require further research. Studies from other surface waters cannot be translated directly to urban canals but provide a basis for further research. For example, studies on the impact of shipping on marine aquatic ecosystems or large rivers identify potential impacts of transportation (Jägerbrand et al. 2019; Que et al. 2020). The impact of TEE in the form of heat extraction and subsequent cold water discharge is hardly studied (Van Megchelen 2017; Harezlak 2021). General knowledge on the ecological impact of changes in water temperature and the impact of transport through filters on aquatic organisms can be translated into hypotheses for research on the impacts of TEE in urban canals (Ellwood et al. 2012; Thackeray et al. 2013; Harezlak 2021; De Jong and Dionisio Pires 2022). Research in non-urban streams showed that bathing activities result in environmental impacts like higher *E. coli* bacteria concentrations, reduced clarity, higher nutrient concentrations, and habitat disturbance (Phillip et al. 2009; Butler et al. 2021). It is likely that these impacts also occur in urban canals. This may be verified with research in urban canals with different environmental background conditions and bathing practices.

## Conclusions

This study provides first insights on how suitability of urban canals and canalized rivers can be improved for transportation, TEE and recreation in the cities of Amsterdam and Ghent. The required alterations for width, depth, and air draft to improve suitability for transportation vary between and within these cities. However, in both cities the entire canal system can become at least suitable for specialized urban freight vessels if bottlenecks in short sections of low suitability are resolved. The canals have fair to excellent suitability for TEE; improving suitability for TEE in most cases requires an increase in either width or discharge. The low suitability for recreation in both cities can only be improved by increasing clarity, often in combination with a reduction of fecal pollution and/or cyanobacteria. These results complement insights from studies in other water types and from studies that focus on factors other than water body characteristics that influence the use potential of urban water. In this study, we analyzed how the SI scores of existing waterways can be increased by adapting parameters that limit suitability. The same methodology can be applied to designs for yet to be created waterways or to be reopened historic canals, in Ghent, Amsterdam and other cities.

## Supplementary Information

Below is the link to the electronic supplementary material.

Supplementary file1 (XLSX 97 kb)

Supplementary file2 (PDF 1067 kb)

## Footnotes

1 www.ec.europa.eu/cip/iee.

2 www.gentsmilieufront.be.

3 www.amsterdamcityswim.nl.

4 www.swiminleiden.nl.

5 http://nwrm.eu/measures-catalogue and https://oppla.eu/nbs/case-studies.
