# Supplementary material for: Improving suitability of urban canals and canalized rivers for transportation, thermal energy extraction and recreation in two European delta cities
Source: Ambio. 2022 Aug 24;52(1):195–209. doi: 10.1007/s13280-022-01759-3 (PMC9666579; doi:10.1007/s13280-022-01759-3)
Supplement: Supplementary file 2 — Supplementary file2 (PDF 1067 kb) [file 13280_2022_1759_MOESM2_ESM.pdf]

Ambio

Supplementary Information
This supplementary information has not been peer reviewed.

Title: **Improving suitability of urban canals and canalized rivers for transportation, thermal energy extraction and recreation in two European delta cities**

Appendix S1: Suitability Indices

Suitability Index for Thermal Energy Extraction (TEE)

The SI score is related to indicative heat extraction capacity, assuming heat extraction during the three warmest months (Van der Meulen et al., 2022). During summer months, when water temperatures are the highest, heat is extracted from surface water with a heat exchanger. Warm water is stored for later use, for example in an Aquifer Thermal Energy Storage in the subsurface. When the heat is needed for heating of buildings during colder months, warm water is pumped up from the storage. Heat is extracted from the water through a heat exchanger and a heat pump further heats the water to the desired temperature for the building's hot water network. Data from the three warmest months are used for calculating the SI score. If the precondition for water depth is met, the SI score is determined by integrating the sub-scores for width, discharge and temperature (Table S1). Because a low value for one parameter can be counteracted by a high value for another parameter to some extent the integration method is the geometric mean of the sub-scores.

Table S1: SI TEE. Criteria for the boundary condition, sub-scores and integration method. n.a.: not applicable. (Table based on Van der Meulen et al., 2022)

Suitability	Sub-score	Parameters			
		Depth [m]	Width [m]	Discharge [$m^3 s^{-1}$]	Temperature [°C]
Excellent	4	n.a.	≥100	≥0.3	≥15
Good	3		10-<100	0.03-<0.3	10-<15
Fair	2		1-<10	0.003-<0.03	5-<10
Low	1		<1	<0.003	<5
Boundary condition:		≥ 0.5[a]	n.a.	n.a.	n.a.
Integration of sub-scores into SI score: geometric mean[b]					

[a] Maximum depth

[b] $SI = (\prod_{i=1}^{n} Si)^{1/n}$ where S_i is the sub-index score of the i-th parameter

Suitability Index for urban freight transportation

The SI score is related to the vessel types that can use the waterway (Van der Meulen et al, 2022). If the precondition for water depth is met, the SI score is determined by integrating the sub-scores for width, depth and air draft (Table S2). Since unsuitable conditions for one parameter cannot be counteracted by another, the minimum operator approach is used for integration of the sub-scores.

Table S2: SI Transport. Criteria for the boundary condition, sub-scores and integration method. n.a.: not applicable. (Table based on Van der Meulen et al., 2022)

Suitability	Sub-Score	Parameters		
		Depth[a] [m]	Width [m]	Air draft [m]
Excellent	4	≥6.3	≥45.6	≥9.1[b]
Good	3	3.1-<6.3	20.2-<45.6	4.0-<9.1
Fair	2	1.4-<3.1	8.4-<20.2	1.7-<4.0
Low	1	0.35-<1.4	<8.4	<1.7
Boundary condition:		≥ 0.35	n.a.	n.a
Integration of sub-scores into SI score: minimum operator[c]				

[a] Minimum depth in fairway

[b] This also applies to open water or movable bridges without air draft constraints

[c] SI=$Min(S_{i=1}^{n})$, where S_i is the sub-index score of the i-th parameter

SI Recreation

The SI score is related to the level of risk for adult swimmers (Van der Meulen et al, 2022). If the precondition for water depth is met, the SI score is determined by integrating the sub-scores for E.coli bacteria (indicator of faecal pollution), Cyanobacteria, pH, clarity and depth (Table S3). Data from the period April-September, the swimming season, are used for calculating the SI score. Since unsuitable conditions for one parameter cannot be counteracted by another, the minimum operator approach is used for integration of the sub-scores.

Table S3. SI Recreation. Criteria for the boundary condition, sub-scores and integration method. n.a.: not applicable. (Table based on Van der Meulen et al., 2022)

Suitability	Sub-Score	Parameters				
		Depth [m]	*E.coli* [cfu/100ml]	Cyano-bacteria [ug/L]	pH	Clarity [m][c]
Excellent	4	Designated bathing zone	<500	<0.5	7-8	>4 or bottom visible
Good	3	≤1.40[a]	500-<1,000	0.5-<12.5	6-<7 or >8-9	2-4
Fair	2	>1.40[a]	1,000-<1,800	12.5-≤75	5-<6 or >9-9.5	1.2-<2
Low	1	n.a.	≥1,800	>75	>9.5 or <5	<1.2
Boundary condition		≥ 0.75[b]	n.a.	n.a.	n.a.	n.a.
Integration of sub-scores into SI score: minimum operator[d]						

[a] 1 m from shore

[b] At deepest point

[c] Secchi-disk transparency depth

[d] SI=$Min(S_{i=1}^{n})$, where S_i is the sub-index score of the i-th parameter

Appendix S2: Used data

The dataset for analyse with all data used for calculating the original sub-scores, SI scores and the impact of raising sub-scores on the SI values per HU or point location is provided in the separate file **vandermeulenetal_datasetS1.xls.** Table S4 contains an overview of all used data sources to create the dataset for calculation of SI scores for TEE, transport and recreation. Where applicable, additional remarks or motivations are provided below.

TEE Amsterdam

- Minimum depth in the fairway: This is an underestimation of the minimum depth in the waterway, but all values are above the precondition of 0.5 m.
- Temperature: data from point locations, at least one measurement per month. At all locations, average temperature and the 95th percentile of decreasing values is above 15°C during the three warmest months, even when taking into account that night-time temperatures are ≤1 °C lower (see Van der Meulen et al., 2022) than daytime values. Therefore, a sub-score of 4 is applied to all HUs.
- Discharge: values generated for line segments in the centre axis of waterways are assigned to the HUs that they cross. The 75th instead of the 95th percentile of decreasing values is used because flow direction variations otherwise result in (near-) zero discharge.
- Width: maximum width is the navigation database is the best available value but will give some overestimation.

TEE Ghent

- Depth: value is derived from allowed ship draft (see section on Transportation). This is an underestimation of maximum depth, but this is no problem because the precondition of 0.5 m is met everywhere.
- Temperature: The dataset includes temperature figures for 42 locations covering many waterways around the city of Gent. Values during the three warmest months are all ≥ 17.3 °C and therefore a sub-score of 4 is applied to all HUs.
- Width: Average width per polygon is determined in ArcGIS by measuring width at several points, the number of points depending on the length and shape of the polygon, and calculating the average.

Transport Amsterdam

- Minimum depth in the fairway: This is an underestimation of the minimum depth in the waterway but all values are above the precondition of 0.35 m.

Transport Ghent

- Depth: value is derived by multiplying allowed ship draft by 1.2 as best estimate of depth. This factor is based on applied keel clearance targets in several local sources (Arcadis, 2007; De Rijck, 2011; Rijkswaterstaat, 2017; https://emis.vito.be/en/node/12231).
- Minimum width: measured by means of Google maps, taking into account jetties and other large permanent structures visible on the satellite image. Like in Amsterdam, some bridges (may) have piers. This seems less frequent pthan in Amsterdam. Their influence on width is not taken into account unless guidance jetties are dividing the fairway.

Recreation Amsterdam

- Water quality data at point locations based on field and laboratory measurements in samples from 0.3 m depth. Except for cyanobacteria, samples are taken at least monthly during summer months.
- We include all locations with at least 3 years of data.

Recreation Ghent

- We include locations with at least 3 days of E.coli- and pH data. At three locations (Houtdok and two locations in Watersportbaan), dozens of datapoints are available for *E.coli* and pH; for the other locations a maximum of 4 (pH) or 5 (E.coli) days of data are available. Apart from the three frequently monitored locations, there are less than 3 days of clarity data available.

(Table S4 on next page)

Table S4. Datasets used for calculating the SI scores. The datasets are not publicly published unless a link to the dataset is provided in this table.

Parameter	Amsterdam		Ghent	
	**Data used**	**Source**	**Data used**	**Source**
**Air draft**	Minimum air draft [m]	Navigation database from municipality Amsterdam[1]	Air draft [m]	Navigation database from VWW (visuris.be). For the recently opened canal Reep: own field measurements[2] and port manager.
**Bridge type**	[fixed/movable]	National navigation map (vaarweginformatie.nl)	[fixed/movable]	Navigation database from VWW (visuris.be)
**Clarity**	Secchi disk depth [m]	Water quality dataset from AGV	Secchi disk depth [m]	Water quality dataset from Stad Gent, Geoloket by VMM
**Cyano bacteria**	Cyanobacteria [μg L^{-1}]	Water quality dataset from AGV	Cyanobacteria blooms notifications	Cyanobacteria blooms notifications register VMM (Blauwalgen — Vlaamse Milieumaatschappij (vmm.be))
**Depth (maximum)**	Depth [m]	Own field measurements [2]	Depth [m]	Greatest value from these sources: VWW (visuris.be), Stad Gent, Geoloket by VMM (http://geoloket.vmm.be/Geoviews/), own field measurements[2]
**Depth (minimum)**	Minimum depth in the fairway [m] for TEE and Transport	Navigation database from municipality Amsterdam[1]	Derived from allowed draft [m]	Navigation database from VWW (visuris.be)
**Depth at 1 m from shore**	Depth [m]	Own field measurements [2]	Depth [m]	Own field measurements[2]
**Designated bathing area status (yes/no)**	[Yes/no]	National bathing water quality register (www.zwemwater.nl)	[Yes/no]	National bathing water quality register (kwaliteitzwemwater.be)
**Discharge**	Values generated for each 15 minutes	Generated by a hydraulic SOBEK model (https://download.deltares.nl/en/download/sobek/) by AGV	Not available	No data available

Table S4 continued

Parameter	Amsterdam		Ghent	
	**Data used**	**Source**	**Data used**	**Source**
E.coli	*E.coli* [cfu100 ml^{-1}]	Water quality dataset from AGV	*E.coli* [cfu100 ml^{-1}]	Water quality dataset from Stad Gent, Geoloket by VMM (http://geoloket.vmm.be/Geoviews/), Big Jump
**Open water** (infinite air draft)	-	Google Maps Satellite images (maps.google.nl)	-	Google Maps Satellite images (maps.google.nl)
**pH**	pH	Water quality dataset from AGV	pH	Water quality dataset from Stad Gent, Geoloket by VMM (http://geoloket.vmm.be/Geoviews/), Big Jump
**Temperature**	Daytime temperature [°C]	Water quality dataset from AGV	Daytime temperature [°C	Water quality dataset from Stad Gent, Geoloket by VMM (http://geoloket.vmm.be/Geoviews/)
**Width (for TEE)**	Maximum width [m] for TEE	Navigation database from municipality Amsterdam[1]	Average width [m]	Measured on base map of teh surface water system from Grootschalig Referentie Bestand Vlaanderen,(www.geopunt.be)
**Width (for Transport)**	Minimum width [m]	Navigation database from municipality Amsterdam[1]	Minimum width [m]	Google maps (https://www.google.com/maps)

[1] Dataset with data from field measurements between 2004 and 2016, data are still valid. Depth and air draft are expressed in a unit that requires correction for water level, which is done with water level data from AGV.

[2] By means of a lead line. For Amsterdam field campaign in January 2021, for Ghent August 2021.

Appendix S3: extra Figures

Figures S1-3 illustrate notable geographic differences with respect to the parameters that need to be altered for improving SI scores.

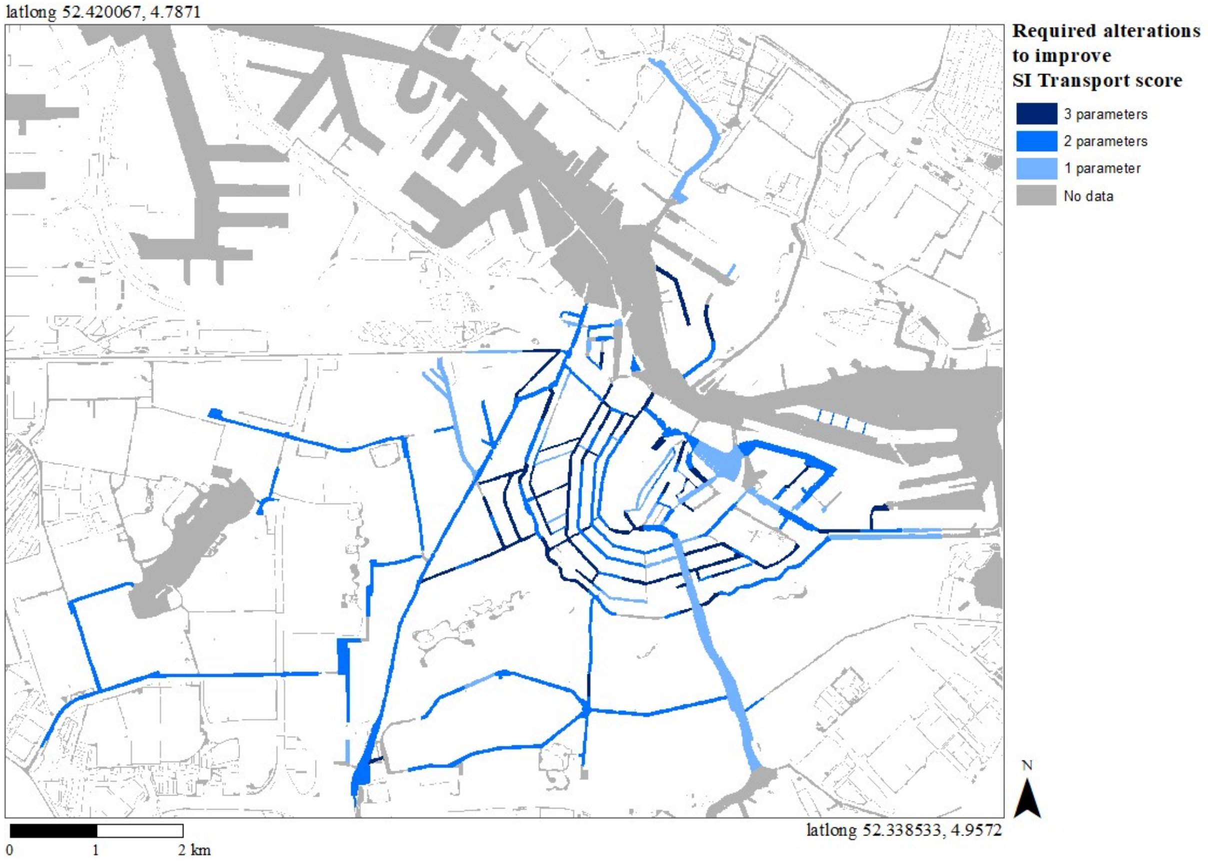

Figure S1 Improving SI Transport scores in Amsterdam requires higher sub-scores for depth, air draft and/or width. In most HUs, two of these parameters need to be altered. HUs where all three parameters need to be improved are mostly located in the city center.

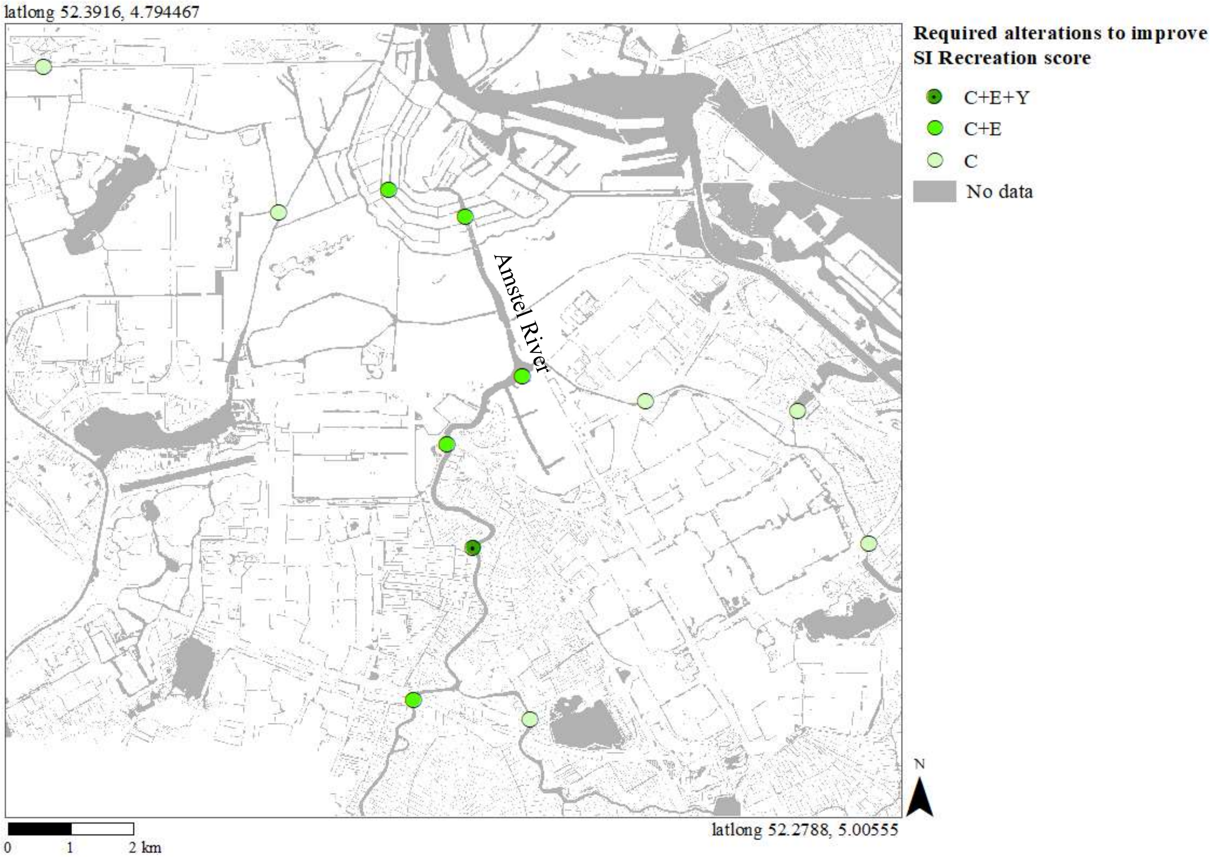

Figure S2 Improving SI Recreation scores in Amsterdam requires higher sub-scores for clarity (C) alone, or in combination with *E.coli* bacteria (E) and Cyanobacteria (Y).

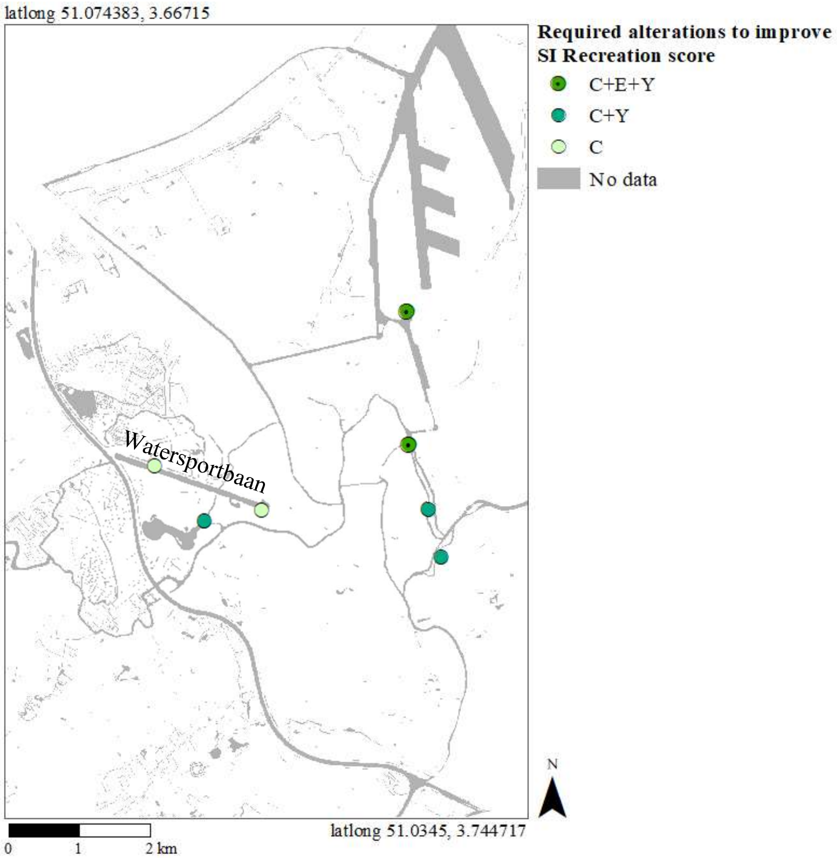

Figure S3 Improving SI Recreation scores in Ghent requires higher sub-scores for clarity (C) alone, or in combination with Cyanobacteria (Y) and *E.coli* bacteria (E).

References

Arcadis. (2007). *Kanaal Gent-Terneuzen: Technische en kostenstudie (met nautische toets) Fase 4*. Retrieved from https://nieuwesluisterneuzen.eu/sites/default/files/downloads/2.2.4%20KGT2008-Onderzoekspakket%201%20Technische%20studie.pdf

De Rijck, T. (2011). *Modellering van het primaire golfsysteem bij binnenschepen.* (Master), Universiteit Gent, Retrieved from https://libstore.ugent.be/

Rijkswaterstaat. (2017). *Richtlijnen Vaarwegen 2017; Kader verkeerskundig vaarwegontwerp*. Retrieved from https://puc.overheid.nl/rijkswaterstaat/doc/PUC_154006_31/

Van der Meulen, E. S., Van Oel, P. R., Rijnaarts, H. H. M., & Sutton, N. B. (2022). Suitability indices for assessing functional quality of urban surface water. *City and Environment Interactions, 13*.
